# Influence on ICU course, outcome and costs for lung transplantation after implementation of the new Swiss transplantation law

**DOI:** 10.1186/2047-1440-3-9

**Published:** 2014-04-01

**Authors:** Stephanie Klinzing, Giovanna Brandi, Dimitri A Raptis, Urs Wenger, Denise Weber, Paul A Stehberger, Ilhan Inci, Markus Béchir

**Affiliations:** 1 Surgical Intensive Care Medicine, University Hospital of Zurich, Zurich, Switzerland; 2 Department of Surgery, Division of Visceral and Transplantation Surgery, University Hospital Zurich, Zurich, Switzerland; 3 Department of Thoracic Surgery, University Hospital Zurich, Zurich, Switzerland; 4 Swiss Paraplegic Centre, Nottwil, Switzerland

**Keywords:** lung transplantation, allocation system, ICU costs

## Abstract

**Background:**

The Swiss organ allocation system for donor lungs was implemented on 1 July 2007. The effects of this implementation on patient selection, intensive care unit course, outcomes and intensive care costs are unknown.

**Methods:**

The first 37 consecutive lung transplant recipients following the implementation of the new act were compared with the previous 42 lung transplant recipients.

**Results:**

Following implementation of the new law, baseline characteristics and cumulative one-year patient survival were comparable in both groups (88.1% vs 83.8%, *P* = 0.58). The costs for each case increased by 35,000 euros after adoption of the new law. Stratifying patients after implementation of the law according to urgency status shows that urgent patients required longer mechanical ventilation (*P* = 0.04), a longer ICU stay (*P* = 0.045) and a longer hospital stay (*P* = 0.04) and ICU costs (median 64,050 euros) were higher compared to regular patients.

**Conclusion:**

The new transplantation law has increased ICU costs with the implementation of the Swiss organ allocation system. Patients listed as ‘urgent’ contribute significantly to the increase in ICU costs.

## Introduction

Since the first heart–lung transplant was conducted in 1980, the option of transplantation has become the standard of care for selected patients with end-stage pulmonary parenchymal or pulmonary vascular disorders [1]. Initially, lung transplantation was an option for patients suffering from pulmonary vascular disorders [2] and cystic fibrosis [3]. Since 1984, all data for heart–lung transplantations have been recorded in the annual registry of the International Society for Heart and Lung Transplantation. Lung transplant data have been included in this register since 1989. The main diagnoses leading to lung transplantation are chronic obstructive lung disease (34.6%), idiopathic pulmonary fibrosis (IPF) (22.6%), cystic fibrosis (16.8%), α1-antitrypsin-deficiency emphysema (6.4%) and pulmonary arterial hypertension (3.2%) [4].

Historically, most transplantation centers used a local allocation system. This strategy is no longer accepted by authorities in most countries worldwide due to its lack of transparency. In addition to increasing the number of donor organs, countries have taken different approaches in establishing allocation systems [5-7]. In Switzerland, as of 1 July 2007 organs have been allocated nationwide and no longer regionally [8]. The Swiss Organ Allocation System (SOAS) for donor lungs is a nationwide, modified chronological system with a defined allocation algorithm and priorities. Priority is given to patients considered as urgent, i.e. those on mechanical ventilation in an intensive care unit (ICU) or on extracorporeal membrane oxygenator [8,9]. Secondary criteria are the medical benefits as defined by experts followed by the length of time waiting [8,9].

Since 2005, the allocation of donor lungs in the United States has been based on the lung allocation score (LAS) [6]. This severity score is based on a model incorporating several factors predicting mortality while on the waiting list as well as post-transplant survival. The LAS is a composite number derived from a formula that takes into account factors from three principal categories. The first factor is the patient’s primary pulmonary diagnosis. The second category relates to disease-specific factors objectifying the disease severity (pulmonary artery pressures, forced vital capacity, oxygen supplementation and ventilator requirement). The third category addresses the patient’s overall health outside of their pulmonary disease (age, body mass index (BMI), New York Heart Association functional class, PCW, 6-min walk distance, diabetes and serum creatinine) [10]. After determining LAS scores for individual patients, available lungs are allocated regionally according to a prioritized ranking based on LAS scores [10].

The effect of the implementation of the new allocation system on lung transplantation in Switzerland in terms of patient selection, ICU course, outcomes and costs are analyzed in this first national single-center study. At our institution for liver transplantation it has been demonstrated that implementation of the MELD-based system (model for end-stage liver disease) led to the selection of sicker patients, increased ICU efforts and higher costs [11].

## Methods

### Patients

This study is a retrospective analysis of all recipients who underwent lung transplantation at the Zurich University Hospital between 1 January 2005 and 31 December 2008. Patients undergoing re-transplantation or combined heart–lung transplantation (*n* = 6) were excluded.

We analyzed the final 42 patients (the pre-group) who had been allocated under the regulations in effect prior to implementation of the new allocation system on 1 July 2007, and the first 37 patients (the post-group) under the new allocation system. In total, 79 lung transplant recipients were included in this study. Following approval by the local ethics board, all patients gave written informed consent prior to transplantation for data analysis after transplantation (TPL).

Demographic variables included age, sex, BMI, creatinine (last value before transplantation), cytomegalovirus status, diagnosis, time on waiting list, location immediately prior to TPL (ICU, ward or at home), need for extracorporeal membrane oxygenation (ECMO) or mechanical ventilation prior to TPL and evidence for pulmonary hypertension assessed using a transthoracic echocardiogram. Pulmonary hypertension was defined as Doppler-calculated pulmonary arterial systolic pressure >50 mmHg at rest [12]. Despite its limitations, this surrogate parameter for characterization of the right heart pre-transplant status had to be used due to the lack of other data in the patient charts.

Operative information collected included whether the patient underwent a unilateral or bilateral lung transplantation, the cold ischemia time, need for intraoperative extracorporeal circulation and transfusion levels of red blood cells, fresh frozen plasma, thrombocytes and fibrinogen.

### Outcomes and survival

Post-operative data collected included the length of stay (LOS) in the ICU, the readmission rate to ICU, post-operative creatinine peak, the incidence and duration of continuous renal replacement therapy (CRRT), the duration of mechanical ventilation and the incidence and duration of ECMO treatment. Primary graft dysfunction, sepsis, multiple organ dysfunction syndrome, rejection, infection, stroke and bowel ischemia were also analyzed.

An analysis of the ICU and in-hospital mortality and 30-day and 12-month cumulative survival was performed. In addition, data were collected on the duration of hospital stay and the incidence of hemodialysis 6 months after transplantation.

### Further analysis and ICU costs analysis

Days in the ICU post-transplantation, and ventilator, CRRT and ECMO days were roughly analyzed using local hospital cost rates for the pre- and the post-groups. Costs are given in euros. To compare the data, a classification of the post-group according to urgency status (SOAS classification) and an approximated LAS score were calculated. Due to missing data (right heart catheterization was not routinely performed), an underestimation of the LAS score was accepted. A stratification of patients according to LAS quartiles was performed analogous to the study performed by Arnaoutakis *et al*. [13], classifying patients into the lower 75% quartiles (Q1 to Q3) (LAS 30.1 to 44.8) and the highest quartile (Q4) (LAS 44.9 to 94.3).

### Statistical analysis

The statistical analysis used SPSS Statistics Version 20 (IBM Corp, 2011). Categorical data were compared with the Fischer’s exact test, continuous variables with the Student’s *t*-test and Mann–Whitney U tests as appropriate. All *P* values were two-sided and statistical significance was considered as *P* < 0.05.

## Results

The baseline characteristics of the lung transplant recipients in both cohorts are shown in Table 1. The baseline characteristics are comparable except for a statistically significant difference in BMI between the two groups (*P* = 0.002). The change in the transplant legislation did not significantly influence the waiting list times 121 (64-243) vs. 162 (69-311) (*P* = 0.56) or the diagnosis (*P* = 0.57). The incidence of ECMO support before TPL with *P* = 0.06 did not reach statistical significance.

**Table 1:** Baseline characteristics

	**Pre (*n*** **= 42)**	**Post (*n*** **= 37)**	***P *** **value**
Women	21 (50.0%)	18 (48.6%)	
Men	21 (50.0%)	19 (51.4%)	
Age (years)	49 (17–69)	54 (13–67)	0.34
Weight (kg)	54 (36–90)	61 (40–100)	0.008
Height (m)	1.65 (1.42–1.83)	1.65 (1.55–1.87)	0.37
BMI (kg/m^2^)	18.9 (14.8–34.0)	22.1 (16.0–61.0)	0.002
Creatinine (μM)	69 (29–96)	61 (31–136)	0.96
CMV positivity	18 (42.9%)	15 (40.5%)	0.84
Pulmonary hypertension^a^	4 (9.5%)	5 (13.5%)	0.58
Diagnosis			0.57
CF	18 (42.9%)	11 (29.7%)	
COPD	9 (21.4%)	10 (27.0%)	
AAT	2 (4.8%)	3 (8.1%)	
IPF	9 (21.4%)	11 (29.7%)	
PAH	1 (2.4%)	0 (0%)	
Other	3 (7.1%)	2 (5.4%)	
Location before TPL			0.26
Home	34 (81.0%)	29 (78.4%)	
Hospital, ward	6 (14.3%)	4 (10.8%)	
Hospital, ICU	2 (4.7%)	4 (10.8%)	
ECMO support before TPL	0 (0%)	3 (8.1%)	0.06
Mechanical ventilation before TPL	1 (4.8%)	4 (10.8%)	0.31

Data expressed as median (range) or number (percentage).

AAT, α1-antitrypsin-deficiency emphysema; BMI, body mass index; CF, cystic fibrosis; CMV, cytomegalovirus; COPD, chronic obstructive lung disease; ECMO, extracorporeal membrane oxygenation; ICU, intensive care unit; IPF, idiopathic pulmonary fibrosis; PAH, pulmonary arterial hypertension; TPL, transplantation.

^a^Assessed by transthoracic echocardiography (pulmonary arterial systolic pressure > 50 mmHg) [8].

There were no significant changes in post-operative ICU data and outcomes (Table 2 and 3) following implementation of the new transplant law. Patients after implementation of the new transplant law had an unchanged LOS in the ICU (median 4 days, inter-quartile range (IQR) 3 to 19, *P* = 0.14) and hospital LOS (median 37 days, IQR 31 to 59, *P* = 0.45). Also the duration of mechanical ventilation remained unchanged at a median of 2 days (IQR 1 to 17, *P* = 0.09) after SOAS implementation. Neither the frequency of CRRT (10% to 19%, *P* = 0.11) nor the frequency of ECMO in the post-operative ICU course (2% to 14%, *P* = 0.26) had statistical significance. Likewise the frequency of ICU complications did not have any significant changes. Overall 1-year survival was 88% before SOAS and 84% after implementation of the new transplant act.

**Table 2:** Operative data

**Intraoperative data**	**Pre (*n*** **= 42) (%)**	**Post (*n*** **= 37) (%)**	***P *** **value**
ECC	17 (40.5%)	18 (48.6%)	0.47
Donor lung			0.63
Unilateral	2 (4.8%)	1 (2.7%)	
Bilateral	40 (95.2%)	36 (97.3%)	
EC (units)	4.6 ± 5.7 (3.0; 0–30)	4.4 ± 4.9 (3.0; 0–17)	0.67
FFP (units)	3.8 ± 6.8 (0; 0–30)	1.7 ± 3.1 (0; 0–10)	0.18
TC (units)	0.5 ± 2.5 (0; 0–16)	0.5 ± 2.0 (0; 0–12)	0.87
Fibrinogen (g)	1.1 ± 2.4 (0; 0–12)	1.7 ± 2.6 (0; 0–8)	0.28
Cold ischemia time (minutes)			
Right	254 (110–404)	240 (127–420)	0.86
Left	336 (170–35)	325 (183–480)	0.91

Data are expressed as median (range), mean ± standard deviation (median; range) or number of patients (percentage).

EC, erythrocyte concentrate; ECC, extra corporeal circulation; FFP, fresh frozen plasma; TC, thrombocyte concentration.

**Table 3:** ICU data

	**Pre (*n*** **= 42)**	**Post (*n*** **= 37)**	***P *** **value**
ICU post TPL (days)	9.5 ± 13.1 (4; 2–66)	17.5 ± 32.5 (4; 2–148)	0.14
Mechanical ventilation post TPL (days)	5.7 ± 9.2 (2; 1–42)	14.6 ± 32.8 (2; 1–148)	0.09
Creatinine peak (μmol/l)	103 (47–395)	95 (56–357)	0.51
CRRT	4 (9.5%)	7 (18.9%)	0.23
CRRT post TPL (days)	1.8 ± 9.6 (0; 0–62)	8.4 ± 31.1 (0; 0–126)	0.19
Readmission to ICU	6 (14.3%)	3 (8.1%)	0.39
ICU stay including readmission (days)	10.1 ± 14.7 (4.5; 2–79)	21.9 ± 42.0 (5; 2–181)	0.10
Mechanical ventilation including readmission (days)	6.2 ± 10.2 (2; 1–45)	18.7 ± 40.1 (2; 1–161)	0.06
CRRT including readmission (days)	2.3 ± 11.6 (0; 0–75)	12.1 ± 37.3 (0; 0–144)	0.11
ECMO	1 (2.4%)	5 (13.5%)	0.05
ECMO after TPL (days)	0.1 ± 0.8 (0; 0–5)	1.5 ± 4.9 (0; 0–22)	0.04
Primary graft dysfunction	5 (11.9%)	6 (16.2%)	0.58
Acute rejection	1 (2.4%)	1 (2.7%)	0.93
Sepsis	3 (7.1%)	6 (16.2%)	0.02
MODS	5 (11.9%)	5 (13.5%)	0.83
Infection	6 (14.3%)	7 (18.9%)	0.58
Stroke	2 (4.8%)	0 (0%)	0.18
Bowel ischemia	2 (4.8%)	4 (10.8%)	0.31
ICU mortality	3 (7.1%)	2 (5.4%)	0.75
Hospitalization days	41 ± 25.6 (36.5; 3–169)	58.2 ± 51.4 (36; 20–270)	0.45
In-hospital mortality	4 (9.5%)	5 (13.5%)	0.58
IHD 6 months post TPL	1 (2.4%)	2 (5.4%)	0.48
Survival 30 days	39 (92.9%)	36 (97.3%)	0.37
Survival 1 year	37 (88.1%)	31 (83.8%)	0.58

Data are expressed as median (range), mean ± standard deviation (median; range) or number (percentage).

CRRT, continuous renal replacement therapy; ECMO, extracorporeal membrane oxygenation; ICU, intensive care unit; IHD, intermittent hemodialysis; MODS, multiple organ dysfunction syndrome; TPL, transplantation.

A rough analysis of ICU costs in terms of ICU, mechanical ventilation (MV), CRRT and ECMO days based on local rates revealed an increase in post-transplant ICU costs averaging 35,000 euros/case after implementation of the new act (Table 4).

**Table 4:** Calculation of ICU costs

	**Pre (*n*** **= 42)**	**Post (*n*** **= 37)**	***P *** **value**
ICU (days; euros)	441; 882,000	810; 162,0000	0.37
Ventilation (days; euros)	263; 132,000	691; 345,500	0.52
CRRT (days; euros)	96; 38,000	437; 174800	0.32
ECMO (days; euros)	5; 5,000	85; 85,000	0.20
Σ ICU costs (euros)	1,057,000	2,225,000	
Σ ICU costs per case (euros)	25,000	60,000	
Difference per case (euros)		35,000	

CRRT, continuous renal replacement therapy; ECMO, extracorporeal membrane oxygenation; ICU, intensive care unit.

For further analysis, the post-transplant group was divided into groups according to the urgency status (SOAS classification) and LAS quartiles as described under Methods. The baseline characteristics of this analysis are presented in Table 5. Analyzing post-operative ICU data and outcome according to the SOAS classification (Table 4) revealed that urgent patients (*n* = 4) required a longer ICU LOS (*P* = 0.045), a longer hospital LOS (*P* = 0.04) and increased duration of MV (*P* = 0.04), while the frequency of CCRT (18% vs 25%) and ECMO (9% vs 50%) did not have statistical significance. One-year survival in the regular group was comparable to urgent patients (82% and 100%, respectively). Analysis of data according to LAS quartiles showed similar results: patients in the highest LAS quartile (*n* = 8) required a significantly longer ICU stay (*P* = 0.01), longer duration of MV (*P* = 0.007) as well as more ECMO days (*P* = 0.02) with comparable 1-year survival between the highest LAS quartile and the other quartiles (86% and 75%, respectively). The rough ICU cost analysis according to SOAS urgency status and LAS quartile is presented in Table 5. While the difference in total ICU costs between regular and urgent patients (median 8,500 euros vs 64,050 euros) did not reach statistical significance (*P* = 0.05), the difference in ICU costs between the lowest 75% LAS quartiles and the highest LAS quartile (median 8,500 euros vs 47,750 euros) was statistically significant (*P* = 0.02).

**Table 5:** Estimation of ICU costs

	**Transplantation law**			**SOAS classification**			**LAS quartile**		
	**Pre (*n*** **= 42)**	**Post (*n*** **= 37)**	***P *** **value**	**Regular**	**Urgency**	***P *** **value**	**Q1 to Q3**	**Q4**	***P *** **value**
ICU (euros)	10,000 (7,000–22,500)	10,500 (7,000–49,000)	0.76	8,000 (6,000–28,000)	39,000 (26,000–61,000)	0.07	8,000 (6,000–10,000)	39,000 (21,000–61,000)	0.02
MV (euros)	1,000 (500–3,500)	1,000 (500–9,000)	0.88	500 (500–6,500)	8,500 (6,500–9,000)	0.06	500 (500–1,000)	8,750 (4,250–13,750)	0.01
ECMO (euros)	5,000 (5,000–5,000)	15,000 (15,000–22,000)	0.67	15,000 (2,000–15,000)	26,500 (22,000–31,000)	0.2	8,500 (2,000–15,000)	22,000 (15,000–31,000)	0.3
**Total ICU costs**	**10,000 (7,000–22,500)**	**10,500 (7,000–49,000)**	**0.82**	**8,500 (6,500–35,400)**	**64,050 (34,500–81600)**	**0.05**	**8,500 (6,500–11,000)**	**47,750 (25,250–102,050)**	**0.02**

Data are median (IQR).

ECMO, extracorporeal membrane oxygenation; ICU, intensive care unit; MV, mechanical ventilation.

## Discussion

This is the first study to evaluate the effect of the implementation of the Swiss Organ Allocation System on ICU outcomes and resource utilization. For this purpose the final 42 patients before and the first 37 patients after implementation of the law on 1 July 2007 were analyzed in this retrospective study.

While baseline characteristics and outcome were comparable between the two cohorts of lung transplant patients, the implementation of the law led to a marked increase in post-transplantation ICU costs of approximately 35,000 euros/case. A further analysis of patients allocated after implementation of the law indicates that treatment for patients listed as urgent according to SOAS differed from patients listed as regular: urgent patients required a five times longer median ICU stay after TPL and twice as long median hospital stay as well as significantly longer mechanical ventilation after TPL. This led to a significant increase in ICU charges: median costs increased by a factor of seven. Patients were stratified into the highest and lowest 75% LAS quartiles for comparison with international data: patients in the highest LAS quartile required a significantly longer ICU LOS and significantly longer duration of mechanical ventilation and ICU costs were significantly higher.

The implementation of SOAS with a change from center-orientated allocation to national allocation did not increase the cold ischemia time of donor lungs, as seen from a comparison of the pre- and post-groups. Since an increased incidence of organ transportation has to be expected with the change in allocation, an eventual effect on cold ischemia time and presumably a negative effect on outcomes have to be considered. Data published by Immer and colleagues for Swisstransplant showed an increased frequency in organ transportation after implementation of the new transplantation act for all organs [14]. For donor lungs, the transplantation frequency at the site of procurement decreased from 21.2% to 7.3% [14]. In agreement with our data, no significant influence on cold ischemia time nationwide was found. Our study did not register the origin of the donor lung, but traditionally the Zurich area has a low organ donor rate [8] so it can be assumed that the percentage of lung transplantations performed with organs donated from a different part of Switzerland remained unchanged and thus there was no change in the cold ischemia time. According to Swisstransplant’s annual report for 2012, 52 lung transplantations were performed in two centers (of which 33 were in Zurich) from 96 multi-organ donors [8]. Overall waiting list times in Zurich were not affected after the implementation of the new transplant act in the period analyzed in this study. Of note, the utility rate of lung donors in Switzerland was 52% in 2012 [8], which is high compared to international data and might influence the early post-operative outcomes and costs.

Concerning the diagnosis leading to transplantation, there was no statistically significant difference between the two cohorts. The second and third priority diagnoses, pulmonary hypertension and IPF, did not have a statistically significant influence. However, there was an increase in lung transplantations performed for IPF from 21.4% to 29.7%. This finding is in accord with international data, which shows that there has been a steady increase in the number of transplant procedures for IPF during the last decade, reaching almost 30% of all procedures performed [4]. The incidence of cystic fibrosis in our small Swiss cohort (overall 37%) is rather high compared to international data (16.8%) [4].

Cost estimations for transplantations are difficult and complex. There are limited data on the estimation of costs for lung transplant patients [15-17]. Our institution recently published our single-center experience concerning liver transplantation after the new transplantation act was implemented [11]. The results show markedly increased costs per transplantation. Those findings were explained because there were higher MELD scores for patients prior to transplantation, causing an increase in pre-transplant costs as well as an increase in post-transplant ICU costs.

The implementation of the high-priority urgent status assigned to patients on invasive mechanical ventilation in the ICU did not have a statistically significant influence after implementation of the new law. However, the analysis of the post-group shows that urgent patients and those in the highest LAS quartile suffered a prolonged transplantation course in terms of LOS in the hospital and ICU and mechanical ventilation. This caused a marked increase in ICU costs.

Arnatoukis *et al*. recently examined the effect of the implementation of LAS on resource utilization and costs [13]. They showed that there was a marked increase in costs for patients in the highest LAS quartile, primarily due to an increase in ICU and hospital days and the duration of mechanical ventilation. Patients with high LAS scores are critically ill [18] with comorbidities. The results of our analysis are in accord with the results of Arnatoukis *et al*. [13]: more critically ill patients proceeding for transplantation leads to an increase in hospital and ICU days, days on mechanical ventilation and overall post-transplant ICU costs. In addition, the trend for the increased use of ECMO is leading to an increase in costs.

The value of ECMO support has been extensively debated since the results of the CESAR trial in 2009 [19]. Similarly, attitudes toward using ECMO for respiratory failure in lung transplant candidates have changed over time. The indication for ECMO is no longer limited to ‘bridge to recovery’ as a desperate maneuver, but has established indications as ‘bridge to transplant’ and ‘bridge to improvement’ [20-22] in the pre-transplant and post-transplant phases, respectively. Internationally, ECMO is being implemented at an earlier stage. Several centers have reported their first experiences with ECMO for non-intubated patients [23,24]. The increased use of ECMO is presumably due to this international development and proven indication.

No difference in 1-year survival was detected between the cohorts for before and after implementation of the law, nor for patients listed as urgent or regular in the post-group. Due to our small sample size, this study might very well be underpowered to detect a difference in survival and the results of a larger cohort study are awaited.

Initial experiences from France with high-emergency lung transplantation showed there was poorer post-transplant survival of these patients compared to regular lung transplant recipients [25], so the further development of the necessity for mechanical ventilation before transplantation has to be assessed.

The main limitation of this retrospective analysis is the low number of patients included, which limits the statistical power of the results.

## Conclusion

In conclusion, the implementation of the new transplant law has so far led to increased ICU costs. In particular, patients listed as urgent seem to have a prolonged post-operative ICU and hospital course.

## Abbreviations

AAT: α1-antitrypsin-deficiency emphysema; BMI: body mass index; CF: cystic fibrosis; CMV: cytomegalovirus; COPD: chronic obstructive lung disease; CRRT: continuous renal replacement therapy; EC: erythrocyte concentrate; ECC: extra corporeal circulation; ECMO: extracorporeal membrane oxygenation; FFP: fresh frozen plasma; ICU: intensive care unit; IHD: intermittent hemodialysis; IPF: idiopathic pulmonary fibrosis; IQR: inter-quartile range; LAS: lung allocation score; LOS: length of stay; MELD: model for end-stage liver disease; MODS: multiple organ dysfunction syndrome; MV: mechanical ventilation; PAH: pulmonary arterial hypertension; SOAS: Swiss Organ Allocation System; TC: thrombocyte concentration; TPL: transplantation.

## Competing interests

The authors declare that they have no competing interests.

## Authors’ contributions

SK analyzed and interpreted the data and drafted the article. DR produced the statistics and undertook a critical review. DW collected the data and undertook a critical review. UW and II interpreted the data and undertook a critical review. PS analyzed the data and undertook a critical review. MB conceived the idea for the study, interpreted the data and undertook a critical review. The submitted version was approved by all authors.
